# Development of High-Level Echinocandin Resistance in a Patient With Recurrent *Candida auris* Candidemia Secondary to Chronic Candiduria

**DOI:** 10.1093/ofid/ofz262

**Published:** 2019-06-01

**Authors:** Mark J Biagi, Nathan P Wiederhold, Connie Gibas, Brian L Wickes, Victoria Lozano, Susan C Bleasdale, Larry Danziger

**Affiliations:** 1 University of Illinois at Chicago College of Pharmacy, Department of Pharmacy Practice; 2 University of Texas Health Science Center at San Antonio, Department of Pathology and Laboratory Medicine, Fungus Testing Laboratory; 3 University of Texas Health Science Center at San Antonio, Long School of Medicine, Department of Microbiology, Immunology, and Molecular Genetics; 4 University of Illinois at Chicago College of Medicine, Department of Medicine

**Keywords:** antifungal resistance, *Candida auris*, candidemia, echinocandin, FKS

## Abstract

**Objective:**

*Candida auris* is a globally emerging pathogen associated with significant mortality. This pathogen frequently is misidentified by traditional biochemical methods and is resistant to commonly used antifungals. The echinocandins currently are recommended as the first-line treatment for C. auris infections. The objective of this work is to demonstrate the challenges associated with C. auris in the real-world setting.

**Methods:**

A 54-year-old male presented to our institution for concerns of sepsis on multiple occasions over a 5-month period. Eleven urine cultures were positive over this timeframe for yeast (9 unidentified Candida isolates and 2 C. lusitaniae isolates). On day 27, the patient developed echinocandin-susceptible candidemia, which was initially identified as C. haemulonii but later accurately identified as C. auris at an outside mycology reference laboratory. Approximately 10 weeks later, the patient had a recurrence of candidemia, this time caused by an echinocandin-resistant C. auris strain.

**Results:**

Genomic DNA sequencing performed at the outside mycology reference laboratory identified a single serine to proline base pair change at position 639 (S639P) in the hotspot 1 region of the FKS1 gene of the echinocandin-resistant strain.

**Conclusions:**

Our experiences highlight 4 major concerns associated with C. auris: misidentification, persistent colonization, infection recurrence despite the receipt of appropriate initial therapy, and development of resistance.

## Teaser

Clinicians should be aware of the diagnostic and therapeutic challenges associated with *Candida auris*. This unique case demonstrates 3 major issues of *C. auris*, misidentification, persistence, and resistance development, in a patient with recurrent *C. auris* candidemia.

## INTRODUCTION

Historically, *Candida albicans* has been the most commonly implicated species in *Candida*-associated infections; however, the prevalence of non-*C. albicans* species has risen and now accounts for roughly 50% of candidemia cases [1–3]. One such species is *C. auris*, which is associated with significant morbidity and mortality, as it frequently is misidentified by traditional biochemical methods and resistant to many common antifungals. The echinocandins generally retain potent in vitro activity against *C. auris* [4] and are recommended as the first-line treatment by the US Centers for Disease Control and Prevention (CDC) [5]. Here, we report our experiences in a patient with persistent candiduria and recurrent *C. auris* candidemia, which was initially misidentified, and who eventually developed high-level echinocandin resistance despite appropriate initial therapy.

## METHODS

**Figure 1.**
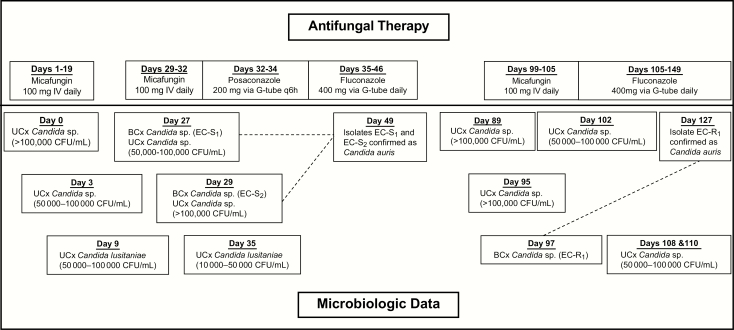
Timeline of Antifungal Therapy and Microbiologic Data.^a^ Abbreviations: BCx, blood culture; EC-R1: echinocandin-resistant strain isolated on day 97; EC-S1: echinocandin-susceptible strain isolated on day 27; EC-S2: echinocandin-susceptible strain isolated on day 29; q6h: every 6 hours; UCx: urine culture. ^a^Patient previously received micafungin at an outside hospital for an unknown duration prior to the initial presentation at our institution (day 0).

A 54-year-old African-American male presented to University of Illinois Hospital and Health Sciences System (UIHHSS) from a nursing home for concerns of sepsis on multiple occasions over a 5-month period with day 0 corresponding to his initial presentation to UIHHSS (Figure 1). His past medical history was significant for quadriplegia, multiple chronic wounds, osteomyelitis of the right hip with abscess formation, and active deep venous thrombosis in the right upper extremity. He had a chronic tracheostomy, chronic indwelling urinary catheter, enteric feeding tube, and colostomy. Prior to his initial presentation, he had a prolonged hospitalization at an outside hospital (OSH), from which he was discharged the day before presenting to our hospital. During hospitalization at the OSH, various wound and intraoperative cultures were positive for *C. glabrata,* carbapenem-resistant *Escherichia coli*, *Lactobacillus* sp., *Proteus mirabilis*, and vancomycin-resistant *Enterococcus.* He was treated and discharged from the OSH with amoxicillin/clavulanate, ciprofloxacin, daptomycin, and micafungin.

During the initial admission to our hospital, cultures obtained on day 0 yielded a single blood culture positive for both coagulase-negative *Staphylococcus* sp. and carbapenem-resistant *Klebsiella pneumoniae* and a urine culture positive for *Candida* sp., which was thought by the primary team to represent colonization. Various imaging studies were suggestive of destructive changes of the right hip with abscess formation, a large decubitus ulcer with extensive bony involvement, and bladder wall thickening suggestive of cystitis. Over the ensuing 3 weeks, the patient remained intermittently febrile and clinically unstable despite the administration of multiple broad-spectrum antimicrobials, including micafungin from days 1–19, which was continued from the OSH for treatment of suspected osteomyelitis (rather than candiduria). All blood cultures during this time were negative, but urine cultures on days 3 and 9 were positive for yeast (Figure 1).

On day 27, the patient became febrile (38.2°C) and 2 days later, micafungin was started when it was reported that 1 of 2 blood cultures were positive for yeast (isolate EC-S_1_). A repeat blood culture from day 29 also was positive for yeast (EC-S_2_). The preliminary report identified EC-S_1_ and EC-S_2_ as *C. haemulonii* by matrix-assisted laser desorption/ionization time-of-flight mass spectrometry (MALDI-TOF MS; Vitek MS [V2], bioMérieux, Marcy I’Etoile, France). Minimum inhibitory concentration (MIC) values were reported on day 33 and are summarized in Table 1. Additionally, urine cultures were positive for a *Candida* sp. on days 27 and 29. Prior to finalization of the susceptibility report of EC-S_1_, micafungin was changed to posaconazole due to concern for a potentially echinocandin-resistant organism given the patient’s recent prolonged exposure to micafungin from days 1–19. Posaconazole was ultimately de-escalated to fluconazole on day 35 based on susceptibilities and the patient was subsequently discharged to a long-term acute care facility (LTAC) on day 36 with an additional 10-day course of fluconazole therapy. Both EC-S_1_ and EC-S_2_ were sent to a mycology reference laboratory (Fungus Testing Laboratory, UT Health San Antonio) for further testing (see Supplementary Data) and were identified as *C. auris*.

**Table 1.** Minimum Inhibitory Concentrations (mg/L) of Isolated *Candida auris* Strains

Strain	AmpB^a^	AFG	CAS	FLU	ISAV^a^	MFG	VRC
EC-S_1_	1	0.12	0.06	2	≤0.03	0.12	0.015
EC-R_1_	1	4	>8	2	≤0.03	>8	0.06

Abbreviations: AFG, anidulafungin; AmpB, amphotericin B; CAS, caspofungin; EC-R_1_, echinocandin-resistant strain isolated on day 97; EC-S_1,_ echinocandin-susceptible strain isolated on day 27; FLU, fluconazole; ISAV, isavuconazole; MFG, micafungin; VRC, voriconazole.

^a^Results for amphotericin B and isavuconazole were obtained based on CLSI M27-A3 (reference standard only) [6]. All other reported values were obtained using YeastOne Sensititre colorimetric assay (TREK Diagnostic Systems, Oakwood Village, OH).

The patient was readmitted to UIHHSS on separate occasions for concerns of sepsis on days 82 and 95, including an admission to the intensive care unit on day 95. Urine cultures remained positive for *Candida* sp. on days 89 and 95, and blood cultures obtained on day 97 were positive for *Candida* (EC-R_1_). The identity of the species of isolate EC-R_1_ was unable to be determined by Vitek MS and was subsequently sent to the reference laboratory. Of note, the patient’s indwelling urinary catheter was exchanged on day 96 but was inappropriately inserted, creating a false passage and subsequent hematuria (this represented the fourth documented urinary catheter exchange at our institution). Micafungin was started on day 99 and changed to fluconazole on day 105 when the susceptibility report of EC-R_1_ revealed an echinocandin-resistant isolate (Table 1). Repeat blood cultures were negative from days 99–114. Meanwhile, multiple urine cultures remained positive for *Candida* during this timeframe as displayed in Figure 1. The patient was discharged on day 120 with indefinite courses of ceftazidime/avibactam, vancomycin, and fluconazole, because of the inability to perform proper source control due to the patient’s refusal for surgical intervention.

On day 127, the reference laboratory reported that EC-R_1_ was identified as *C. auris*. DNA sequencing performed on the EC-S and EC-R isolates identified a single serine to proline base pair change at position 639 (S639P) in the hotspot 1 region of the *FKS1* gene (see Supplementary Data).

## DISCUSSION

Our case highlights 4 key points for clinicians regarding *C. auris*: misidentification, persistence, recurrent, and the development of resistance.

The misidentification of *C. auris* by various traditional biochemical methods has been well documented and recommendations for accurately identifying *C. auris* are available from the CDC [7]. In our case, 15 *Candida* isolates (11 urine and 4 blood) were cultured from our patient and sent to our institution’s clinical microbiology lab for identification by MALDI-TOF MS. Eleven isolates were unable to be identified and 2 isolates were reported as either *C. haemulonii* (EC-S_1_ and EC-S_2_) or *C. lusitaniae*, which are both closely phylogenetically related to *C*. *auris* [8, 9]. Both bloodstream isolates initially reported as *C. haemulonii* were later properly identified as *C. auris*. None of the remaining isolates of unspeciated *Candida* or *C. lusitaniae* were sent for further identification testing; however, we believe these isolates represent un- or misidentified *C. auris* isolates. Current recommendations by the CDC only recommend further workup for *C. auris* when either *C. haemulonii* or no identification is reported by Vitek MS (in vitro diagnostic [IVD] library only) but not *C. lusitaniae* [10]. However, in the official decision summary report of the US Food and Drug Administration review of Vitek MS v3.0 it is stated that, “*Candida auris* is not currently in the knowledge base; testing of this species will usually give no identification but may also result in a misidentification as either *Candida haemulonii* or *Candida lusitaniae*” [11]. Additionally, we also were able to identify at least 2 additional reports where *C. auris* was misidentified as *C. lusitaniae* by Vitek MS [12, 13]. Misidentification of *C. auris* can lead to delays in implementation of proper infection control and prevention measures. In our case, contact precautions in a private room were rapidly implemented due to the patient’s history of carbapenem-resistant Enterobacteriaceae, but these precautions differ from those at our institution for *C. auris*, which call for additional measures, such as daily cleaning of high-touch areas with bleach. *C. auris* is a reportable disease to the department of public health. When *C. auris* was confirmed, we performed surveillance screening cultures in collaboration with the Chicago Department of Public Health. We did not find any evidence of transmission within our institution; however, these cultures were performed after the patient was discharged from our hospital.

Among the distinguishing challenges that complicate the management of patients with *C. auris* is the striking ability of this pathogen to cause persistent colonization or infection, or both [12, 14, 15]. In a recent study including 11 patients with 2 or more positive urine cultures obtained at least 1 day apart, the average time between the first and last reported *C. auris* urinary isolates was 49.5 days (range 1–259) [16], demonstrating the remarkable ability of this pathogen to cause persistent colonization. We believe that our patient already was colonized with *C. auris* when he initially presented to our institution, considering his positive urine culture on day 0 and the fact that he represented the first documented case of *C. auris* at our institution, making it unlikely that he contracted *C. auris* during his hospital stay. Although the patient did receive micafungin until day 19 to complete the course of therapy for suspected fungal osteomyelitis initially diagnosed at the OSH, all subsequent antifungal therapy was started either empirically or as culture-directed therapy for *C. auris candidemia*. Furthermore, the patient had significant exposure prior to his initial presentation to our institution to various healthcare settings, broad spectrum antibiotics and antifungals, and additional predisposing characteristics, such as the presence of urinary and central venous catheters, chronic wounds, immunosuppression, and respiratory insufficiency requiring mechanical support; all of these had been previously reported as risk factors associated with *C. auris* [17, 18]. Although candiduria does not commonly progress to candidemia [3], a number of similar cases describing patients with proven or suspected *C. auris* urinary colonization preceding candidemia have been previously reported [13, 16, 19, 20]. Furthermore, episodes of recurrent candidemia reported both here and elsewhere are likely attributable to cases of persistent colonization [13, 15].

The widespread resistance to fluconazole, which generally is considered the drug of choice for *Candida* urinary tract infections (UTIs), further complicates the management of *C. auris* candiduria [3]. Alarmingly, in our case, candiduria persisted following the completion of fluconazole therapy for the initial episode of candidemia despite in vitro susceptibility, a phenomenon previously encountered in at least 1 other patient [15]. This raises a particularly concerning question as to how to manage patients with persistent *C. auris* candiduria considering its widespread resistance to fluconazole, reports of fluconazole failure against infections caused by susceptible isolates, and poor urinary penetration of other azoles and the echinocandins. Flucytosine previously has been recommended as an option for *C. auris* UTIs [21], and at least 1 institution has reported using combination therapy with amphotericin B and flucytosine for suspected *C. auris* UTIs, although no clinical outcomes were reported [22]. Clinical experience with flucytosine is otherwise extremely limited, but its use for *C. auris* UTIs warrants consideration given that primary resistance is uncommon [18], it is predominantly excreted into the urine in its active form [23], and currently is recommended as a preferred agent for symptomatic fluconazole-resistant *C. glabrata* cystitis [3]. The use of amphotericin B bladder irrigations is controversial [24] and had largely fallen out of favor in clinical practice, but there has been a renewed interest in this approach in recent years [25–28]. Clinical experience with *C. auris* certainly is lacking, but clinicians should consider the use of amphotericin B bladder irrigations for persistent amphotericin B-susceptible *C. auris* infections limited to the lower urinary tract given the extremely limited treatment options.

As previously stated, antifungal-resistance among *C. auris* is common, as demonstrated by resistance rates of approximately 90% and 30% for fluconazole and amphotericin B in the US, respectively [7]. Fortunately, echinocandin-resistance is relatively uncommon making these drugs suitable first-line agents. However, clinicians should be aware that echinocandin-resistant *C. auris* isolates have been encountered in at least 5 other patients in the US, leading the CDC to make the following statement, “Based on these findings, CDC is concerned that echinocandin-resistant *C. auris* could become more common” [29]. Resistance to echinocandins previously has been reported to occur in patients with *C. auris* isolates initially echinocandin-susceptible [22, 29]. We believe that although the episodes of candidemia in our patient were separated by 10 weeks, both instances were caused by the same *C. auris* strain, which developed the S639P point mutation in the hotspot 1 region of *FKS1* due to prolonged exposure to micafungin and potentially subinhibitory urine concentrations that may have promoted the development of resistance [30–34]. This mutation previously has been reported in echinocandin-resistant *C. auris* isolates and corresponds to known mutations connected with echinocandin resistance in *C. albicans* (S645P) and *C. glabrata* (S629P) [35]. We verified that the patient completed the course of fluconazole therapy prescribed at discharge on day 36 and were able to confirm that no other antifungals were administered at the LTAC between hospitalizations. Additionally, no other echinocandin-resistant *C. auris* isolates had been reported in Illinois (personal communication, Illinois Department of Public Health), making it unlikely that echinocandin-resistance was acquired through transmission of a separate strain.

Based on our experience reported here, we have the following recommendations regarding the 4 key points highlighted by our case: misidentification, persistence, recurrence, and the development of resistance. In addition to current recommendations advising further testing of *C. haemulonii* and unidentified *Candida* isolates, institutions that utilize MALDI-TOF MS technology also should perform further identification testing on isolates of *C. lusitaniae*. This recommendation is only applicable to institutions not utilizing the recently developed MALDI-TOF MS databases, such as the Bruker Maldi Biotyper CA System (Bruker Daltonics Inc., Billerica, MA) or the research use only (RUO) library for Vitek MS, both of which have been updated to accurately identify *C. auris* [13, 36, 37]. Additionally, clinicians should consider further identification testing of *C. haemulonii*, *C. lusitaniae*, and unspeciated *Candida* isolates from nonsterile sites in select patients, including those who are critically ill or have risk factors for *C. auris*. Even if the suspicion of a true clinical infection is low, early identification of *C. auris* allows for rapid implementation of infection control measures to decrease the likelihood of patient-to-patient transmission and a potential subsequent outbreak. Next, urinary catheters should be removed as soon as feasibly possible in patients with *C. auris* candiduria. For patients with symptomatic *C. auris* candiduria, although the optimal treatment is unknown, clinicians should be aware that, despite fluconazole’s high urinary tract penetration, it is a poor choice due to widespread resistance and reports of treatment failures in patients with susceptible isolates. Because of limited treatment options, amphotericin B administered intravenously or as a bladder irrigation (lower UTIs only) with or without flucytosine should be considered as a potential treatment option for susceptible isolates in this setting; future studies investigating this practice are warranted. Finally, although there are currently no approved susceptibility breakpoints for *C. auris*, we recommend performing repeat susceptibility testing for patients with persistent or recurrent infections who have previously received an echinocandin due to the possibility that this pathogen may be capable of developing de novo *FKS1*-mediated resistance following echinocandin exposure.

Altogether, our experiences reported here add to the growing literature on the alarming concerns and issues associated with *C. auris*, including misidentification, persistent colonization, infection recurrence despite the receipt of appropriate initial therapy, and development of resistance.

## Supplementary Data

Supplementary materials are available at *Open Forum Infectious Diseases* online. Consisting of data provided by the authors to benefit the reader, the posted materials are not copyedited and are the sole responsibility of the authors, so questions or comments should be addressed to the corresponding author.

ofz262_suppl_supplementary_material
