# Supplementary material for: Development of High-Level Echinocandin Resistance in a Patient With Recurrent Candida auris Candidemia Secondary to Chronic Candiduria
Source: Open Forum Infect Dis. 2019 Jun 1;6(7):ofz262. doi: 10.1093/ofid/ofz262 (PMC6602379; doi:10.1093/ofid/ofz262)
Supplement: ofz262_suppl_supplementary_material [file ofz262_suppl_supplementary_material.docx]

**Supplementary Methods**

***Species Identification.*** For molecular identification portions of the *C. auris* cultures that had been suspended in Buffer G2 (Qiagen, Valencia, CA) were lysed using a bead beater instrument (Precellys Evolution, Bertin Instruments, Rockville, MD), followed by the addition of proteinase K and incubation at 56°C. The DNA was then extracted using an EZ1 DNA tissue kit with a BioRobot EZ1 instrument (Qiagen). The internal transcribed spacer region (ITS) and D1/D2 rRNA gene were then amplified by PCR using previously described primers [1-3]. The PCR products were then sequenced, assembled, and analyzed using Sequencher software version 5.4.6 (Gene Codes, Ann Arbor, MI). The sequences were queried in GenBank using the BLASTn algorithm at the NCBI website ([www.ncbi.nlm.nih.gov](http://www.ncbi.nlm.nih.gov)). Both the ITS and D1/D2 sequences showed 100% similarity to *C. auris* for isolates EC-S_1_ and EC-R_1_ (ITS match to GenBank Accession Nos. KT305974, KT305968, and KT305984 with 398/398, 398/398, and 397/397 base pair [bp] matches, respectively; D1/D2 match to GenBank Accession Nos. KU886679 and KU886678, with 562/562 bp matches for each isolate). Growth was also observed following incubation at 42°C for each isolate.

***Antifungal Susceptibility Testing.***  Initial antifungal susceptibility testing was performed by the YeastOne Sensititre colorimetric assay, and results were confirmed at the outside microbiology lab using the methods described in the Clinical and Laboratory Standards (CLSI) M27-A3 reference standard [4]. MIC values were measured after 24 hours of incubation at 35°C. MIC values for amphotericin B and isavuconazole were only performed based on CLSI recommendations. For amphotericin B, the MIC was read as the lowest concentration of drug that resulted in complete inhibition of growth. For the other agents, the MIC was the lowest concentration that resulted in at least 50% inhibition of growth compared to the growth control.

***FKS Sequence Analysis.*** Primers to the putative *C. auris FKS1* hotspot 1 and hotspot 2 regions were designed by first identifying the *FKS1* gene from the *C. auris* genome sequence. A BLASTp search of the *C. auris* genome sequence was conducted using the *C. albicans* Fks1p protein sequence (GenBank Accession No. XP_721429). The *C. auris* reference sequence (XM_018312389) that corresponded to the *C. albicans* *FKS1* sequence was downloaded into MacVector (MacVector, Inc., Apex, NC) and translated. The translated sequence was then searched with the *C. albicans* Fks1p hotspot 1 sequence (FLLSLRD) to identify the corresponding *C. auris* DNA region, which could then be used for primer design. Forward and reverse primers (Eurofins Genomics, Inc., Louisville, KY) (Table S1) were identified in the *C. auris FKS1* coding sequence (CDS) (bp 1738-2152) with MacVector and used to amplify a 414 bp fragment from EC-S_1_, EC-R_1_, and CBS10913, which served as a control isolate. The fragments were purified (Qiaquick PCR Purification Kit, Qiagen, Inc., Valencia, CA) and sequenced with the same primers (Eurofins Genomics). The *C. auris* hotspot 2 region was identified using the same strategy based on the *C. albicans* hotspot 2 amino acid sequence (DWIRRYTL). Forward and reverse primers (Table S1) spanning bp 3944-4205 of the CDS were identified and used to amplify a 261 bp region from each strain, which was sequenced as above.

The putative *C. auris* Fks1p homolog listed as hypothetical protein QG37_02962 (GenBank Accession No. XM_018312389) displayed 84% identity to the *C. albicans*protein. The predicted protein was 1888 amino acids in length with a molecular weight of 216.69 kD. A search of the protein revealed the two conserved Fks1p hotspots. Hotspot 1 (FLTLSLRDP) was found at amino acids 635-643 and hotspot 2 (DWIRRYTL) was found at amino acids 1350-1357. Both hotspots displayed 100% conservation compared to the *C. albicans* Fks1p hotspots. Sequence analysis of both hotspot PCR products from all three strains revealed that there were no differences in hotspot 2 from each when compared to the GenBank reference sequence. For hotspot 1, there was also 100% identity with the *C. albicans* hotspot 1 sequence, however EC-R_1_ had a single serine to proline base pair change at position 639 (S639P). The hotspot 1 amino acid sequence of the GenBank Fks1p reference sequence was FLTL**S**LRDP (which matched both EC-S_1_ and CBS10913), however the hotspot 1 sequence of EC-R_1_ was FLTL**P**LRDP.

**Supplementary References**

1. White TJ, Bruns T, Lee S, Taylor J. Amplification and direct sequencing of fungal ribosomal RNA genes for phylogenetics. In: Innis MA, Gelfand DH, Sninsky JJ, White TJ. PCR protocols: a guide to methods and applications. San Diego, CA: Academic Press, **1990**:315-22.
2. Romanelli AM, Sutton DA, Thompson EH, Rinaldi MG, Wickes BL. Sequence-based identification of filamentous basidiomycetous fungi from clinical specimens: a cautionary note. J Clin Microbiol **2010**; 48(3): 741-52.
3. Kurtzman CP, Robnett CJ. Identification of clinically important ascomycetous yeasts based on nucleotide divergence in the 5' end of the large-subunit (26S) ribosomal DNA gene. J Clin Microbiol **1997**; 35(5): 1216-23.
4. CLSI. Reference method for broth dilution antifungal susceptibility testing of yeasts; Approved standard - Third Edition. Wayne, PA: Clinical and Laboratory Standards Institute, **2008**.

**Table S1.** Primers used for *FKS1* sequencing.

| **Primer** | **Region Sequenced** | **Sequence** |
| --- | --- | --- |
| C.auris.HS1.F | *FKS1* hot spot 1 | 5’-CCTTTGGGTGGCTTGTTCAC-3’ |
| C.auris.HS1.R | *FKS1* hot spot 1 | 5’-TGTTTCTCCATGGGGTCAAGA-3’ |
| C.auris.HS2.F | *FKS1* hot spot 2 | 5’-TGAACTCTTTGGCCCACGAA-3’ |
| C.auris.HS2.R | *FKS1* hot spot 2 | 5’-AGGAACACTTCGAACATCGGA-3’ |
